# Thy-1 (CD90)-regulated cell adhesion and migration of mesenchymal cells: insights into adhesomes, mechanical forces, and signaling pathways

**DOI:** 10.3389/fcell.2023.1221306

**Published:** 2023-11-30

**Authors:** Alejandra Valdivia, Ana María Avalos, Lisette Leyton

**Affiliations:** ^1^ Division of Cardiology, Department of Medicine, Emory University, Atlanta, GA, United States; ^2^ Instituto de Ciencias Biomédicas, Facultad de Ciencias de la Salud, Universidad Autónoma de Chile, Santiago, Chile; ^3^ Cellular Communication Laboratory, Programa de Biología Celular y Molecular, Center for Studies on Exercise, Metabolism and Cancer (CEMC), Instituto de Ciencias Biomédicas (ICBM), Facultad de Medicina, Universidad de Chile, Santiago, Chile; ^4^ Advanced Center for Chronic Diseases (ACCDiS), Faculty of Chemical and Pharmaceutical Sciences & Faculty of Medicine, Universidad de Chile, Santiago, Chile

**Keywords:** reactive astrocytes, cancer cells, fibroblasts, signal transduction, adhesome, mechanobiology, integrins, Thy-1 (CD90)

## Abstract

Cell adhesion and migration depend on the assembly and disassembly of adhesive structures known as focal adhesions. Cells adhere to the extracellular matrix (ECM) and form these structures via receptors, such as integrins and syndecans, which initiate signal transduction pathways that bridge the ECM to the cytoskeleton, thus governing adhesion and migration processes. Integrins bind to the ECM and soluble or cell surface ligands to form integrin adhesion complexes (IAC), whose composition depends on the cellular context and cell type. Proteomic analyses of these IACs led to the curation of the term adhesome, which is a complex molecular network containing hundreds of proteins involved in signaling, adhesion, and cell movement. One of the hallmarks of these IACs is to sense mechanical cues that arise due to ECM rigidity, as well as the tension exerted by cell-cell interactions, and transduce this force by modifying the actin cytoskeleton to regulate cell migration. Among the integrin/syndecan cell surface ligands, we have described Thy-1 (CD90), a GPI-anchored protein that possesses binding domains for each of these receptors and, upon engaging them, stimulates cell adhesion and migration. In this review, we examine what is currently known about adhesomes, revise how mechanical forces have changed our view on the regulation of cell migration, and, in this context, discuss how we have contributed to the understanding of signaling mechanisms that control cell adhesion and migration.

## 1 Introduction

Cell migration is essential during homeostatic and pathological processes in multicellular organisms. Cells can migrate individually or collectively but their movement is rarely random; thus, migration is directional and cells move commanded by the myriads of cues that surround them (Shellard and Mayor, 2020). In addition, cellular events occurring during migration, such as cell adhesion, polarity, and mechanical strain, are tightly regulated in a spatiotemporal fashion by these environmental cues, which include the type, amount, and properties of the extracellular matrix (ECM) surrounding the cells (Yamada et al., 2019; Yamada and Sixt, 2019; Doyle et al., 2022). Cells interact with the ECM through integrin receptors to promote cell migration (Wu et al., 2017).

Integrins are α and β transmembrane heterodimers that bind ECM proteins, as well as cell receptors and soluble ligands. In humans, at least 18 α and 8 β subunits have been described, generating 24 heterodimers, which recognize and interact with specific ligands. Integrin activation relies on conformational changes triggered after interacting with their ligands (outside-in) and cytoplasmic tail-binding partners (inside-out) (Huttenlocher and Horwitz, 2011).

**FIGURE 1:**
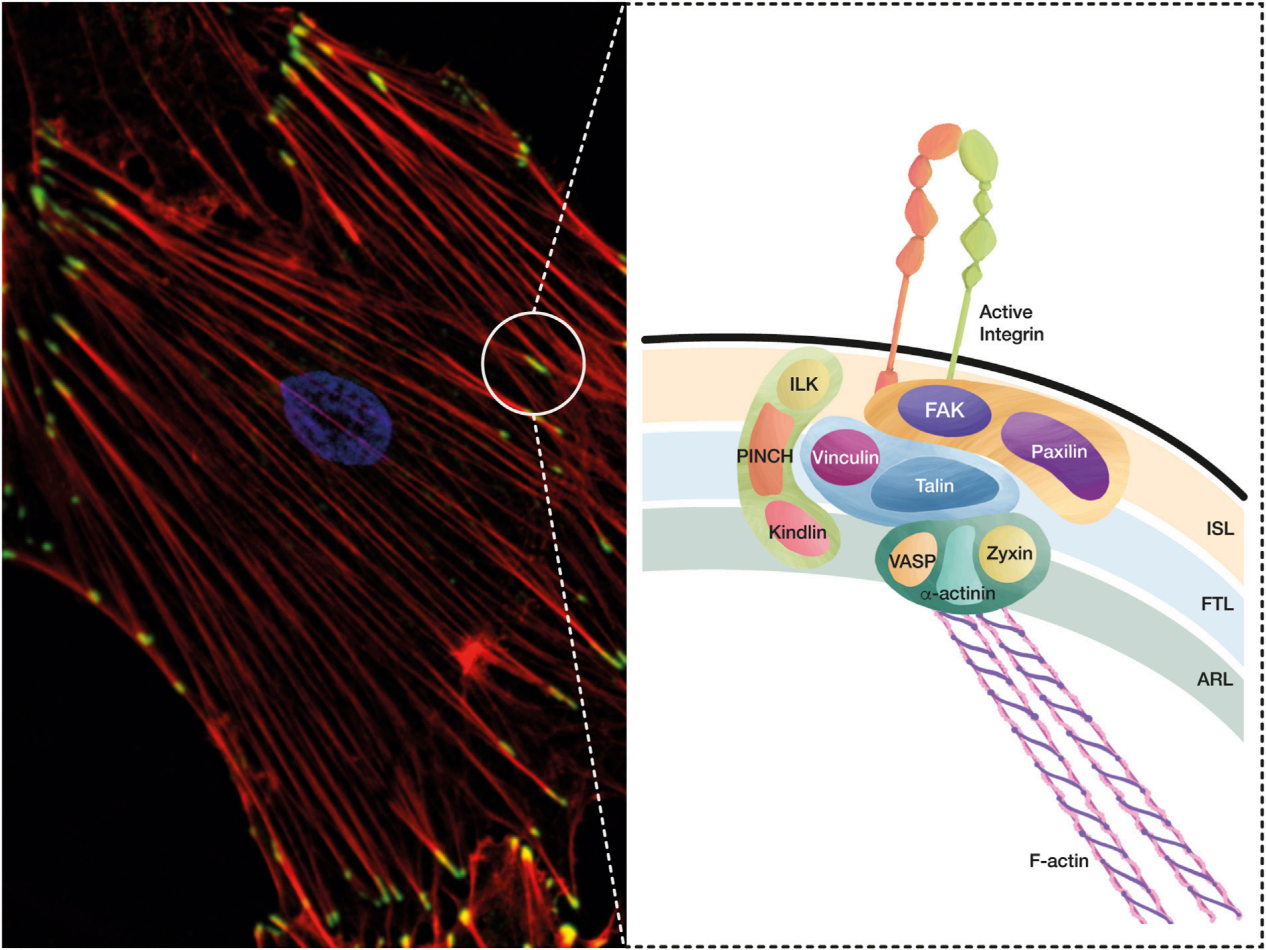
A conceptual map of the adhesome. The integrin adhesome is a protein complex that has been defined through careful evaluation of several proteomic analyses of IACs. These complexes formed by integrin-ligand interaction lead to FA and SF formation, which are essential structures in cell adhesion and migration, and play a key role during mechanotransduction. **(A)** The microphotography shows FAs in green (Vinculin staining, Alexa488), SFs in red (Phalloidin, Alexa594), and the nucleus in blue (DAPI). **(B)** Based on the interactions and functions of IACs during integrin adhesion and signaling, a consensus integrin adhesome has been curated into four nodes, which contain: i) ILK–PINCH–Kindlin, ii) FAK–Paxillin, iii) Talin–Vinculin, and iv) α-actinin–Zyxin–VASP. These protein nodes organize in three interconnected layers: i) the integrin signaling layer (ISL) that contains integrin tails, Paxillin, and FAK; ii) the force transducing layer (FTL) that includes the mechanotransducer proteins Talin and Vinculin, which are the physical link between integrins and the filamentous actin (F-Actin); and iii) the actin regulatory layer (ARL) that contains actin-regulatory proteins, such as VASP, Zyxin, and α-actinin.

The initial engagement of integrins with the ECM forms Nascent Adhesions (NAs), which are small dot-like structures (∼0.25 μm diameter) composed of around 50 integrin dimers that serve as platforms for the recruitment and activation of numerous proteins (Changede et al., 2015; Henning Stumpf et al., 2020). NAs are transient structures with a short lifetime (∼1 min) that primarily form in the lamellipodium of migrating cells. Even though actin polymerization is necessary for the formation of NAs, they form and persist independently of the activity of non-muscle Myosin IIA. NAs that remain assembled grow to form Focal Contacts (FCs). These FCs are larger in size (1 μm), localize in the lamellipodium-lamella interface, and their formation depends on myosin activity. FCs are transient structures that display a 1–2 min lifetime and then mature into larger and elongated structures known as Focal Adhesions (FAs) (Figure 1). FAs exhibit lifetimes of several minutes and a broad spectrum of sizes (1 μm W x 3–5 μm L) due to their dynamic maturation process. Actin crosslinking mediated by α-actinin and Myosin IIA mediates the initial maturation of FAs, which directly bind to the actomyosin cytoskeleton to transduce cellular strain. Contractile actin microfilament bundles and Myosin crosslinked by several proteins form Stress Fibers (SFs) (red staining, Figure 1), which are involved in cell adhesion, migration, and mechanotransduction. SF contractility can either help FA maturation or promote their disassembly (Echtermeyer et al., 2001; Oakes et al., 2012; Burridge and Guilluy, 2016; Livne and Geiger, 2016).

Cell migration requires the integration and coordination of specific FA dynamics at the front, center, and rear of the cells. Migrating cells continuously form and disassemble their adhesions at the leading edge. FAs mature at the front edge and towards the side and back of the cells. Then, actin SFs attached to FAs contract and retract the rear end, and lastly, adhesions at the tail disassemble, resulting in forward movement. This process of constant assembly/disassembly of FAs is termed adhesion turnover (Webb et al., 2002). FA turnover is tightly regulated in a spatiotemporal manner by actin depolymerization and reorganization (Alexandrova et al., 2008; Shemesh et al., 2009), microtubule dynamics (Small et al., 2002), Calpain proteolysis (Franco and Huttenlocher, 2005), integrin endocytosis (Laukaitis et al., 2001; Broussard et al., 2008; Ezratty et al., 2009), and mechanical tension (Crowley and Horwitz, 1995; Wolfenson et al., 2011), among others. Thus, the front-to-rear polarity that governs cell migration is tightly regulated by many signaling pathways and feedback mechanisms that control cytoskeletal and FA dynamics.

Hundreds of proteins have been described within mature FAs. These proteins constitute “The Cell Adhesome”, composed of many types of proteins: adhesion receptors, signaling proteins, enzymes (protein kinases, phosphatases, and proteases), and cytoskeletal, adapter, and scaffold proteins, which interact with many others, thus forming large and complex networks (examples and references in Table 1) (Zaidel-Bar et al., 2007a). When multiple adhesome components are mutated or dysregulated, these adhesive structures mediating cell–ECM interactions lead to abnormal migration and invasion, which have been implicated in pathological conditions, such as cancer and cardiovascular diseases (Winograd-Katz et al., 2014; Byron et al., 2015).

**TABLE 1:** General functions, classification, gene name, and binding partner’s examples of proteins forming mature focal adhesions.

Focal adhesions (mature adhesions)			
	General functions	Classification	Gene name (binding partner’s number) and examples*
**Kindlin**	Involved in inside-out activation of integrins (Montanez et al., 2008; Moser et al., 2008; Ussar et al., 2008; Rognoni et al., 2016)	Cytoskeletal Adapter Mechanotransducer	**FERMT1 (33):** β1, β3, β6 integrin, ILK, Plk1, Migfillin, Kindlin-2, PI(3,4,5)P3, PI(4,5)P2
			**FERMT2 (171):** β1, β2, β3 integrin, Clathrin, ILK, β-Catenin, Rac, Sos1, Migfillin, Kindlin-1, Smad3, PI(3,4,5)P3, PI(4,5)P2

	Force on ***Syndecan-4***, together with EGFR, activates Kindlin-2-integrin, leading to RhoA activation (Chronopoulos et al., 2020)		**FERMT3 (58):** β1, β2, β3 integrin, RACK1, PI(3,4,5)P3, PI(4,5)P2
**Talin**	Involved in inside-out activation of integrins (Wolfenson et al., 2013; Sun et al., 2019)	Cytoskeletal Adapter Mechanotransducer	**TLN1 (232):** β1, β2, β3, β5, β7 integrin, Actin, Paxillin, Vinculin, Kindlin, DLC1
	Links integrins with Actin filaments and participates in assembly of Actin filaments (Chen et al., 1995; Calderwood and Ginsberg, 2003; Jiang et al., 2003; Klapholz et al., 2015)		**TLN2 (44):** Rac2, Zyxin

	Tensile force applied on ***Syndecan-4*** induces Talin recruitment (Chronopoulos et al., 2020)		
**FAK**	Phosphorylates targets such as Paxillin and cortactin during FA dynamics (Schaller, 2010; Tomar et al., 2012; Tapial Martinez et al., 2020; Le Coq et al., 2022)	Tyrosine Kinase Mechanotransducer	**PTK2 (367):** αv, β1, β3, β4 integrin, Paxillin, Src, Fyn, Lck, CAS, Grb2, ROCK1, Talin, Akt
	Mechanical strain can activate FAK (Li et al., 1997; Seong et al., 2013; Bauer et al., 2019)		
	***Thy-1*** induces FAK phosphorylation on Y^397^ (Leyton et al., 2001; Rege et al., 2006; Kong et al., 2013; Avril et al., 2017)		
	***Syndecan-4*** modulates FAK phosphorylation (Wilcox-Adelman et al., 2002; Valdivia et al., 2020)		
**Paxillin**	Links the ECM to the actin cytoskeleton and transduces mechanical cues (Zaidel-Bar et al., 2007b; Parsons et al., 2010; Widmaier et al., 2012)	Cytoskeletal scaffold Actin binding	**PXN (368):** α4, αv, β1, β3 integrin, Actin, CSK, Fyn, ILK, PAK, Src, ROCK1, FAK, Talin, VASP

	***Thy-1*** induces the recruitment of paxillin to FAs (Leyton et al., 2001)		
	Recruits ***Syndecan-4*** to FAs by binding syndesmos (Denhez et al., 2002)		
**Integrins**	Mechanoreceptors that link the ECM with the cytoskeleton. They sense tensile force and transduce it into chemical signals to affect cell function (Hynes, 2002; Campbell and Humphries, 2011; Widmaier et al., 2012; Kechagia et al., 2019; Kolasangiani et al., 2022)	Cell adhesion receptors	**ITGB1 (394)**: ILK, ICAM, VCAM, FAK, Paxillin, Talin, Cdc42, Rac1-3, RhoA, B, C, D, F, G, H, J, Q, U, and V, Kindlin 1–3, α-actinin, Collagen, Fibronectin
	They recruit hundreds of proteins to their cytoplasmic domains to form anchoring points for the cell with their substrate (Zaidel-Bar et al., 2007a; Winograd-Katz et al., 2014)		**ITGB3 (94):** ILK, FAK, Akt1, VEGFR2, PECAM1, Thy-1, Talin, FGFR1, PDGFRA-B, PDK1, Src, Vimentin, Fibronectin, Vitronectin
	They cooperate with ***Syndecan-4*** to form FAs (Couchman and Woods, 1999; Saoncella et al., 1999; Bass et al., 2007; Araki et al., 2009; Morgan et al., 2013; Nikmanesh et al., 2019)		
**ILK (Integrin-like kinase)**	ILK is recruited to β1 and β3 integrin cytoplasmic tails and may indirectly associate with actin through Parvin and Paxilin (Fukuda et al., 2009; Choi et al., 2012; Vakaloglou and Zervas, 2012; Widmaier et al., 2012; Elad et al., 2013)	Pseudokinase Scaffold	**ILK (323):** β1, β2, β3, α5, αv integrin, Caveolin-1, Paxillin, Akt1, Cdc42, Rac1, RhoA, F, J, Q, T and V, Csk, Ect2, Fibronectin

**α-Parvin**	Parvin is part of the ILK signaling axis	Cytoskeletal	**PARVA (76):** Actin, ILK, Coronin 1b, Kindlin 1–3, Paxillin, Rac1-2, RhoQ
	Binds to Actin through CH domains. Associates with actin filaments (Olski et al., 2001; Legate et al., 2006; Vakaloglou and Zervas, 2012; Ain and Firdaus, 2022)		**PARVB (19):** ILK, ARHGEF6, Kindlin3, ParvinA, RhoF
			**PARVG (49):** ILK, Cadherin3
**α-actinin**	Links integrins to the actin cytoskeleton	Cytoskeletal Actin binding Actin crosslinking	**ACTN1 (351):** Palladin, FAK, Src, Zyxin, Ect2, ICAM1 and 5, RhoD and F, Talin, VASP
	NA growth is accompanied by recruitment of α-actinin (Bois et al., 2006; Gardel et al., 2010; Roca-Cusachs et al., 2013; Legerstee et al., 2019)		

	Links ***Syndecan-4*** to the actin cytoskeleton (Greene et al., 2003; Okina et al., 2012)		
**Vinculin**	Anchors F-actin to the membrane in cell-cell and cell-ECM contacts (Parsons et al., 2010; Carisey and Ballestrem, 2011; Legerstee et al., 2019; Austin et al., 2023)	Cytoskeletal adapter Actin binding	**VCL (283):** α4, α5, β1 integrin, α-actinin, Actin, Coronin2B, FAK, Paxillin, Src, Talin, VASP, Caveolin, VEGFR2, FGFR1, LPP

	Most prominent protein marker of FAs		
	Concentrates at sites of fast-growing ends of actin filaments		
	Interacts with α-actinin (Jockusch and Isenberg, 1981; Bois et al., 2006; Carisey and Ballestrem, 2011; Thievessen et al., 2013)		
	***Thy-1*** induces Vinculin recruitment into FAs (Leyton et al., 2001)		
	***Thy-1*** reduced expression decreases Vinculin levels (Lee et al., 2013)		
	***Syndecan-4*** stimulation induces Vinculin recruitment to FAs (Bellin et al., 2009; Cavalheiro et al., 2017)		
**Zyxin**	Concentrates in FAs, cell-cell junctions, and actin SFs (Crawford and Beckerle, 1991; Vasioukhin et al., 2000; Yoshigi et al., 2005; Hansen and Beckerle, 2006; Sperry et al., 2010; Legerstee et al., 2019)	Cytoskeletal Zinc-binding Adapter	**ZYX (322):** α-actinin, Actin, ARHGEF 6, 9, 10, 19, and 39, Ajuba, VASP, ARHGAP 5, 9, 11b, 21, 31, 32, and 36, Kindlin-2, Paxillin, Talin, Vinculin


	Acts as a mechanotransducer and can regulate gene expression (Yoshigi et al., 2005; Smith et al., 2013; Wang et al., 2019)		
	Localizing VASP in FAs. Indirectly regulates Actin assembly by binding VASP (Reinhard et al., 1995; Drees et al., 2000; Fradelizi et al., 2001)		
**VASP**	Promotes actin polymerization by interacting with the profilin:G-actin complexes and the free barbed ends of F-actin, facilitating the transfer of monomeric Actin to the barbed end, and preventing the activity of capping proteins (Huttelmaier et al., 1998; Bear et al., 2002; Barzik et al., 2005; Hansen and Mullins, 2010)	Cytoskeletal Actin polymerization factor	**VASP (310):** Actin,LPP, Zyxin, Akt1, Cdc42, MRTFA, Grb2, Vinculin, α-actinin, Kindlin-2, α4 integrin, Palladin, Pard3, ZO-1 and 2, VCAM1

**Myosin II**	Generates mechanical force. Mechanical strain generated by Myosin II stimulates FA maturation and eventually, disassembly (Wakatsuki et al., 2003; Clark et al., 2007; Vicente-Manzanares et al., 2009; Pasapera et al., 2010)	Cytoskeletal Actin-based motor protein	**MYH2 (46):** α-actinin, IQGAP3, Tropomyosin-1, Supervillin

	***Thy-1*** increases the levels of MLC phosphorylation on serine 19 (Perez et al., 2021)		

(*) Interaction information was obtained from the National Center for Biotechnology Information (NCBI) and the National Library of Medicine (NLM) (https://www.ncbi.nlm.nih.gov/gene). Interacting partners were identified by affinity capture, co-fractioning, two-hybrid, affinity capture-MS, and proximity label-MS. We show the gene name and the number of interaction partners (#) described for the human gene, followed by some examples of binding partners that participate in cytoskeletal dynamics. CH: calponin homology, ECM: extracellular matrix, NA: nascent adhesion, FA: focal adhesion, SF: stress fiber.

Cells migrate in response to an enormous amount of cues, which have been separated in diverse categories according to the type of signal [reviewed in (Shellard and Mayor, 2020; SenGupta et al., 2021)]. The best described modes of cell migration are chemotaxis (soluble chemical cues), haptotaxis (immobilized cues), and durotaxis (mechanical cues). In chemotaxis, soluble chemical ligands bind to membrane receptors that concentrate at the cell front, thus leading to polarization of signaling molecules (SenGupta et al., 2021). During haptotaxis, gradients of immobilized ligands in extracellular sites are detected by molecules in FAs, promoting signals that are like those triggered by chemical signals (King et al., 2016). In durotaxis, mechanical forces exerted by ECM rigidity (Stephens et al., 2008; SenGupta et al., 2021), also known as stiffness, or pulling forces exerted by cell-cell interaction, transduce mechanical signals in a process known as mechanotransduction (Rens and Merks, 2020). Overall, all these signaling pathways, induced by different cues, translate into modifications of the actin cytoskeleton that regulate directional cell migration.

ECM proteins are the most typical integrin ligands but not the only ones. Other ligands include growth factor receptors that regulate cell proliferation, differentiation, and migration; as well as other cell surface receptors that mediate cell-cell communication (Brizzi et al., 2012; LaFoya et al., 2018). The latter category includes the integrin receptor (ligand) identified by our group, Thy-1, also known as CD90 (Leyton et al., 2001). Thy-1 is a cell adhesion molecule that contains a single Arg-Gly-Asp (RGD)-like peptide, which binds and activates different integrins, thus favoring communication between cells. Thy-1 also possesses a heparin-binding domain (HBD), which activates Syndecan-4, an equally important player of the cell migration process (Valdivia et al., 2020). Importantly, evidence reported by our group has contributed to identifying two participants of the integrin adhesome: Thy-1 as a cellular receptor for integrins (Leyton et al., 2001; Zaidel-Bar et al., 2007a), and the cell polarization protein Partition-defective 3 (PAR-3) (Valdivia et al., 2020). We initially described Thy-1 as the binding partner of integrin β3 (Leyton et al., 2001), and subsequently, Thy-1 was categorized by Geiger and collaborators as an associate component of the avb3 integrin adhesome (Zaidel-Bar et al., 2007a), whereas PAR-3 was reported to interact with Syndecan-4 to control FAK phosphorylation (Valdivia et al., 2020). Additionally, the interactions between Thy-1 and the mechanoreceptors integrin and Syndecan-4 led us to explore the effect of force on Thy-1-induced effects (Burgos-Bravo et al., 2020; Perez et al., 2021), which we discuss in this review.

In this article, we also describe the general structure and organization of adhesomes, we portray mechanobiology as an important regulator of adhesion and migration, and finally, we contrast and discuss how 20 years of our research converged and helped to elucidate new mechanisms in the context of cell adhesion and migration mediated by the interaction of Thy-1 with αvβ3 integrin and Syndecan-4.

## 2 Adhesomes: a growing list of proteins participating in adhesion and migration

### 2.1 The integrin adhesome

The term adhesome was first used by Richard Hynes in 2006 to describe the cluster of cell-cell and cell-matrix adhesion receptors in an organism (Whittaker et al., 2006). Benny Geiger and coworkers then expanded this concept to include the entire network of structural and signaling proteins that regulate the adhesion of cells to the ECM (Zaidel-Bar et al., 2007a; Zaidel-Bar and Geiger, 2010; Winograd-Katz et al., 2014). Since the major cell-matrix adhesion receptors are integrins, the adhesome containing this type of receptor is currently known as the “integrin adhesome” and contains cytoskeletal proteins recruited to integrin cytoplasmatic tails, which mediate integrin signaling functions (Winograd-Katz et al., 2014; Horton, 2021). Therefore, integrins and the signaling emanating from these receptors are the central components of the adhesome.

An integrin adhesome composed of 2,412 proteins was generated by integrating several integrin adhesion complex (IAC) proteomes (Chastney et al., 2021). Different biochemical approaches and rigorous curation of seven available data sets helped define a consensus integrin adhesome composed of 60 core proteins, such as Palladin, Vinculin, Paxillin, FAK, Tensin, and VASP (Horton et al., 2015). Of these 60 proteins, 42 were clustered into four nodes based on their interactions and previous functional implications in integrin adhesion and signaling: i) ILK–PINCH–Kindlin, which links signaling of receptor tyrosine kinases to integrins, ii) FAK–Paxillin, a node that facilitates FA turnover, iii) Talin–Vinculin, which provides a link between the FA complex and the cytoskeleton, and iv) α-actinin–Zyxin–VASP, which connects SFs to integrins through Vinculin (Horton et al., 2015) (Figure 1). This core integrin adhesome fulfills central functions of integrin adhesions: linkage of the ECM to F-actin, regulation of F-actin organization and dynamics, and signal transduction. The other 18 proteins did not have reported functions at FAs or interactions with other adhesome members (Han and de Rooij, 2016).

Decades of investigations have revealed that the large repertoire of FA proteins found in integrin adhesomes organizes at the nanoscale level in three interconnected layers or modules (Kanchanawong et al., 2010) running parallel to the cell membrane, rather than in three independent layers. Advances in three-dimensional super-resolution fluorescence microscopy techniques revealed these three distinct nanolayers, where integrins and actin are vertically separated by a ∼40 nm FA core region consisting of multiple protein-specific strata: i) the integrin signaling layer (ISL) lies closest to the adherent membrane (within ∼10–20 nm) and contains the cytoplasmic tails of integrins that interact with the signaling proteins Paxillin and FAK; ii) the actin-regulatory layer (ARL) is located approximately 50 nm below the ISL, associates with actin SFs, and contains actin-modulating proteins, such as Zyxin, VASP, and α-actinin; and iii) the force transducing layer (FTL) lies in between the ISL and ARL, and contains Talin and Vinculin (and possible interactors), which form a physical link that transmits force between integrins and the actin cytoskeleton (Kanchanawong et al., 2010; Case and Waterman, 2015; Xia and Kanchanawong, 2017) (Figure 1). Each of these three nanolayers contains the main proteins found in three of the four integrin adhesome interaction nodes mentioned above. The head domain of Talin interacts with the cytoplasmic tail of integrins, while its C-terminal rod domain binds F-actin, and these interactions physically link the ISL and ARL (Calderwood and Ginsberg, 2003; Yao et al., 2014). On the other hand, integrins and associated complexes segregate laterally into nanoclusters with distinct integrin activities and mechanical properties (Shroff et al., 2007; Spiess et al., 2018). As reported, the binding of proteins, such as Paxillin, VASP, Zyxin, and Vinculin, to FAs is dynamically regulated according to FA orientation (FAs aligned to the long-axis or perpendicular to the cell edge) and location (FAs close to or away from the cell edge) (Legerstee et al., 2019). Additionally, the composition of the nanolayers in FAs is very dynamic, and could constantly change depending on various factors, such as the cell microenvironment, ECM stiffness, cell contractility, or signaling due to phosphorylation/dephosphorylation of many proteins (Manninen and Varjosalo, 2017; Tang et al., 2018). These dynamic changes in the organization of FAs and their components can affect different cellular responses, including cell adhesion and migration.

### 2.2 Regulation of adhesomes by protein domains

Adhesomes are enriched in various protein domains, such as the Pleckstrin homology (PH) and FERM domains, which target proteins to the plasma membrane; the Calponin homology (CH) domain, which binds to F-actin and plays essential roles in cytoskeletal dynamics and signaling; the Src homology 2 (SH2) domain, which interacts with phosphorylated tyrosine residues; and the armadillo (ARM) and LIM domains, which mediate specific protein-protein binding (Liu and Lauffenburger, 2009). One of the most important domains found in FA proteins is the SH2 domain. Tyrosine-specific kinases and phosphatases within FAs regulate the phosphorylation status of their substrates, creating docking sites for proteins with SH2 domains (Pawson et al., 2001). The transient localization of tyrosine-specific kinases and phosphatases within FAs probably plays a key role in dynamically controlling the association of other SH2 domain-containing FA proteins within FAs and thus, regulates the composition of FAs and FA-mediated signaling. For instance, FAK signaling results from its autophosphorylation on Tyr-397 (pY^397^) in response to integrin-mediated adhesion, allowing interactions with multiple SH2-containing signaling proteins that bind to this site, including Src, PI3K, Grb7, PLCγ1, and Nck-2 (Wu et al., 2015). Src is a non-receptor protein tyrosine kinase that contains an N-myristoylation site, as well as SH3, SH2, and kinase domains (Kaplan et al., 1994). Once recruited, Src phosphorylates two FAK-interacting proteins, Crk-associated substrate (CAS) and Paxillin, which regulate the activation of Rho-family GTPases controlling cell motility (Hanks et al., 2003). Among the phosphatases within FAs, the SH2-domain-containing inositol 5′-phosphatases (SHIPs), SH2-domain-containing PTP2 (SHP2), and SH2-domain-containing protein tyrosine phosphatase substrate 1 (SHPS1) have been found to bear SH2 domains that are required for locating in proximity to their target proteins. These phosphatases decrease the number of docking sites for proteins recruited to FAs and inactivate kinases (Saxton et al., 2000; Sattler et al., 2001). Of note, many other phosphatases, such as PP2A, SAP1, PTP-PEST, and PTP1B, are targeted to FAs to regulate adhesion and migration (Larsen et al., 2003; Frijhoff et al., 2014). In the case of LIM domains, the biochemical and structural nature of most of the proposed LIM domain-mediated interactions has not been precisely defined yet. However, many proteins containing multiple LIM domains are recruited to FAs under mechanical tension, such as LIMK1 and LIMK2, which stabilize the actin cytoskeleton by phosphorylating and inactivating Cofilin (Schiller and Fässler, 2013; Sun and Alushin, 2022). Therefore, LIM domains were postulated as tension sensors and localizers, targeting proteins to specific subcellular locations, such as tensioned or injured F-actin networks (Schiller et al., 2011; Smith et al., 2013).

These examples of regulation by protein domains present in many components of the adhesome are helping us to understand the structural interactions required for the physiological functioning of FAs at the level of their assembly, mechanosensing, and signal transduction and how these processes control cell migration (Ripamonti et al., 2021).

### 2.3 Regulation of adhesomes by molecular switches

The interactions between the various adhesome components regulate two major functions of adhesion sites: first, their role as “scaffolds”, which allows the physical interaction between the ECM-bound cell and the cytoskeleton, leading to the assembly of tissues with a particular structure and mechanical properties; and second, their “signaling” activity, through which cells can sense the chemical and mechanical properties of the external environment and respond accordingly by activating signaling pathways that regulate cell structure, dynamics, behavior, and fate (Winograd-Katz et al., 2014). More than half of the links interconnecting different adhesome components can be switched on or off by signaling elements, and some of these regulated interaction switches are conformational switches, GTPase switches, lipid switches, proteolytic switches, and phosphorylation switches (kinase/phosphatase balance). During conformational switches, adhesion proteins can be found in a folded, inactive state, and after binding to a lipid or a GTPase, being phosphorylated/dephosphorylated, or suffering mechanical strain, they change conformation into an open, active state that exposes new binding sites (Zaidel-Bar et al., 2007a). Integrins are a well-known example of a conformational switch because they thermodynamically fluctuate between different conformations. They are kept latent in a low-affinity conformation by inhibitory molecules. Endogenous inhibitory proteins like shank-associated RH domain-interacting protein (SHARPIN) and mammary-derived growth inhibitor (MDGI) maintain the bent conformation of integrins by binding to the cytoplasmic tail of the α integrin subunit to inhibit β1 integrin (Bouvard et al., 2013). Other inhibitory proteins are integrin cytoplasmic-associated protein-1 (ICAP-1) and Filamin A. ICAP-1 inactivates β1 integrins, while Filamin A binds to β1, β3, and β7 integrin cytoplasmic tails, stabilizing their bent conformation [reviewed by (Bouvard et al., 2013; Pang et al., 2023)]. Integrins then switch to a high-affinity conformation after interacting with the ECM. This high-affinity conformation is stabilized by mechanical forces transduced via Talin and Kindlin. Tensional forces induce integrin conformational changes both outside (headpiece extension) and inside the cell (leg opening) (Puklin-Faucher and Vogel, 2009; Thinn et al., 2018) and thereby, promote ligand binding and recruitment of adapter proteins in the cytoplasm to strengthen the integrin and actomyosin linkage. Therefore, adhesion is further stabilized by the formation of catch bonds (characterized by an increase in the lifetime of the bond after increasing the force applied) and integrin clusters (Askari et al., 2009; Sun et al., 2019; Kolasangiani et al., 2022). Each of the three adhesome vertical layers or modules mentioned in Section 2.1 contains mechanosensitive proteins that can undergo conformational changes or post-translational modifications under tensile forces, thus leading to changes in force transduction and/or induction of biochemical signaling, such as activation of FAK, Src, and Rho family GTPases (Sun et al., 2016).

The Rho family of small GTPases are also considered molecular switches that cycle between an “off” state (GDP-bound) and an “on” state (GTP-bound). Moreover, their activity is regulated by GTPase-activating proteins (GAPs) that accelerate their intrinsic GTPase activity through guanine nucleotide exchange factors (GEFs) that induce the release of GDP to allow their activation, or by guanine nucleotide dissociation inhibitors (GDIs), which stabilize the inactive GTPases (Arthur et al., 2002; Burridge and Wennerberg, 2004).

Among the lipid switches, the most studied is the PI(4,5)P2 to PI(3,4,5)P3 switch catalyzed by PI3K. Phosphoinositides differentially distribute in migrating cells, where PI(4,5)P2 localizes at the leading edge and PI(3,4,5)P3 at the rear end. In particular, PI(4,5)P2 has been related to FA turnover at the leading edge (Ijuin, 2019; Posor et al., 2022). Additionally, phosphoinositides participate in the oligomerization of receptors, such as the heparan sulfate proteoglycan Syndecan-4, an event necessary for the activation of its downstream signaling (Oh et al., 1997).

Lastly, one of the best-studied examples of a proteolytic switch is the cysteine protease Calpain. Calpain is activated by increased calcium concentration and cleaves various proteins of the integrin adhesome. Calpain is involved in FA turnover at the rear end by inducing proteolysis of FA proteins, such as Talin, FAK, Paxillin, and Vinculin (Chan et al., 2010; Cortesio et al., 2011; Ono and Sorimachi, 2012). Therefore, Calpain could represent a mechanism for regulating FA turnover, which is important in processes like cell adhesion, spreading, and migration (Franco et al., 2004; Wells et al., 2005; Rodriguez-Fernandez et al., 2021).

Overall, processes such as cell adhesion and migration are cyclically regulated and tightly coordinated by the linked interconnections occurring within the adhesome, where several of the proteins involved are switched on and off to maintain the dynamics of the process.

### 2.4 Regulation of cell migration by FA dynamics

FA dynamics or turnover refers to the cyclic assembly and disassembly of FAs. In migrating cells, FA turnover is precisely controlled by the coordination of several signaling pathways and the modulation of tensile forces (Broussard et al., 2008). The initial assembly of FAs is mediated by actin polymerization and treadmilling, which occurs either at the edge of the lamellipodium or at the tip of the invading protrusion. Application of mechanical stress by the treadmilling of actin to nearby adhesion molecules (mostly integrins and associated components, such as Talin, Vinculin, and FAK/PYK2) then triggers the assembly of functional adhesion structures (Revach et al., 2020). Integrin engagement, combined with the local tensile force, is essential and sufficient to activate these mechanosensitive adhesome components, resulting in the development and growth of FCs into FAs. The subsequent assembly and growth of an “adhesion plaque” is enriched with actin-binding adhesome components (including Talin and Vinculin) and also augments outward pressure by acting on the leading edge of the lamellipodium, thus driving cell migration (Revach et al., 2020). These specific events in FA dynamics occurring in different subcellular locations create a complex adhesion and migration machinery that is essential for cellular function.

Evidence has shown that FA disassembly is promoted by the cleavage of FA proteins in the rear end, protein phosphorylation, and the dissociation of adapter proteins (Parsons et al., 2000; Legerstee and Houtsmuller, 2021; Mishra and Manavathi, 2021). Other modes of FA disassembly involve microtubules, which traditionally have been considered key to triggering FA turnover, due to the crosstalk between microtubules and actin. Forces within FAs may exert feedback on microtubules to complete FA turnover, and *vice versa* (Burridge and Guilluy, 2016; Gupta et al., 2016). The evidence indicates that polymerizing microtubules have a role in the activation of the Rho family member Rac, which in turn promotes actin polymerization, membrane protrusion, FA turnover, and migration (Seetharaman and Etienne-Manneville, 2019). As reported in astrocytes, acetylated microtubules control the distribution and dynamics of FAs and the release of GEF-H1 from microtubules to the cytosol, where it activates RhoA-actomyosin contractility and generates forces to promote collective migration of astrocytes (Chang et al., 2008; Seetharaman et al., 2022). Therefore, as for the actin cytoskeleton, microtubules also play a crucial role in FA dynamics.

Indeed, pioneering live imaging experiments showed that microtubules use actin SFs as tracks to direct themselves to the vicinity of FAs (Kaverina et al., 1998). This microtubule guidance along SFs requires the crosslink protein MACF1/ACF7 to reach FAs (Wu et al., 2008). Microtubules are then captured and stabilized around FAs through their interaction with KANK1 and Talin, which in turn, bind to actin (Rafiq et al., 2019). Microtubules retract and regrow to contact FAs several times until FAs reach full maturation (Seetharaman and Etienne-Manneville, 2020). Fully mature FAs are then “sensed” by microtubules, which deliver autophagosomes to the FAs, and concomitantly retract (Kenific and Debnath, 2016). Autophagosomes contain LC3, which recognizes the cargo receptors NBR1 and c-Cbl that interact with Paxillin when phosphorylated by Src (Sharifi et al., 2016; Chang et al., 2017). These interactions activate signaling events involved in the removal of phosphorylated FA components that may lead to FA disintegration (Sandilands et al., 2011; Lu et al., 2021). Although the process of autophagy to selectively engulf FAs is poorly understood, it represents another mode of FA dynamics regulation requiring the communication of FAs with microtubules.

Another study in lung microvascular endothelial cells revealed that manipulation of β3 integrin expression leads to β3 integrin-dependent changes in microtubule behavior (Atkinson et al., 2018). According to these authors, engagement of αvβ3 integrin with fibronectin (FN) at mature FAs localizes a Rcc2/Anxa2/Rac1 complex to these sites to actively destabilize microtubules (perhaps by controlling its exposure to GEFs). Interestingly, when αvβ3 integrin was not present, the complex associated instead with α5β1 integrin and exerted the opposite effect on microtubules. This re-positioning of Rac1 activity means that this small GTPase plays a role in microtubule-linked endothelial cell migration only when αvβ3 integrin is not present in mature FAs (Atkinson et al., 2018).

Another group of cytoskeletal elements that interplay with actin to control FA maturation is septins [see review in (Cavini et al., 2021)]. Septins are GTP-binding proteins that form oligomeric complexes, which further assemble into filaments, gauzes, and rings that interact with the membrane and the cytoskeleton. Knockdown of SEPT2 or SEPT9 in migrating MDCK cells led to a higher number of smaller FAs closer to the cell edge (Dolat et al., 2014). Although assembly and disassembly rates of NAs were the same as in control cells, the stabilization phase was abolished in SEPT2 knockdown cells, resulting in short-lived FAs that failed to mature (Dolat et al., 2014). SEPT2 knockdown in cancer-associated fibroblasts was also reported to lead to fewer FAs, whereas SEPT2 or SEPT9 knockdown in murine and human melanoma cells led to a decrease in FA size (Calvo et al., 2015; Farrugia et al., 2020). How septins affect FA maturation and, thus the lifetime of FAs is still not clear since septins are largely excluded from FAs, as shown by immunostainings in NRK, MDCK, and U2OS cells. The role of septins in FA stabilization is most likely due to their association with FA-anchored SFs. The current hypothesis is that septins, through their actin filament cross-linking activity, maintain the integrity and organization of SFs, which in turn are required for the stabilization and maturation of FAs (Livne and Geiger, 2016).

Therefore, the evidence revealed that timely FA turnover and directed cell migration depend on the interplay between many molecular factors beyond microtubules (Mavrakis and Juanes, 2023).

### 2.5 Advances in the understanding of how integrin adhesomes regulate migration

The main challenges in studying the molecular functionality of adhesomes have been: i) the molecular heterogeneity of integrin adhesions within and between different cellular systems, ii) the large number of adhesome components, iii) the participation of multiple “functional switches” that can turn adhesome components “on” or “off”, and iv) the overall dynamic nature and plasticity of the adhesion system as a whole (Winograd-Katz et al., 2014). To add to this complexity, the repertoire of ECM components (collectively known as the “matrisome”), is rather wide and includes over 1,000 “core” and “associated” components (Naba et al., 2016). Many integrin-associated proteins and their differential interaction with the plasma membrane form a puzzle with 200 to 1,000 different pieces, for which there is only limited structural and biochemical information. Furthermore, many of these adhesome proteins undergo conformational changes under tensional stress, thus increasing the intricacy of adhesion sites (Bachmann et al., 2019). Due to the underlying complexity of the matrisome and adhesome, it has been historically challenging to undertake mass spectrometry-based profiling for the characterization of the ECM and IACs because of the low affinity and transient nature of the molecular interactions occurring at these sites. Despite these difficulties, the use of cell-permeant chemical cross-linkers improved the recovery of IAC proteins bound to either FN-coated microbeads or plastic dishes (Schiller et al., 2011; Horton et al., 2015). These advances led to the characterization of IACs from several cell types. Even though there is a consensus adhesome, the composition and stoichiometry of the meta-adhesome depend on the cell type being analyzed, the integrin-receptor repertoire expressed by that cell type, the turnover of FAs, and the experimental conditions that are used. Recent technical, methodological, and bioinformatic advances in proteomics have shown promise in characterizing the matrisome and topology of adhesome networks in both health and disease (Krasny and Huang, 2021). In addition, tight communication between the matrisome and adhesome controls the cytoskeleton and thus, cell migration [for more details, see (Krasny and Huang, 2021)].

Cell adhesion receptors like Thy-1 form part of the associated components of the adhesome due to their interaction with α and β integrin subunits, which are intrinsic adhesome components (Leyton et al., 2001; Zaidel-Bar et al., 2007a). Our group was the first to report the presence of a single RGD-like peptide in a conserved sequence of the human, rat, and mouse Thy-1 polypeptide (Leyton et al., 2001). The tripeptide found in Thy-1 was an RGD-like peptide (RLD), which recognizes the αvβ3 integrin present in astrocytes (Leyton et al., 2001; Hermosilla et al., 2008). This association, which reportedly occurs with many other integrins (Leyton et al., 2019), was recognized as part of the dense connectivity of the adhesome network (Zaidel-Bar et al., 2007a), and was also backed up by our findings, reporting that FAs formed due to Thy-1/integrin binding contain many proteins described in IACs [(see Section 2.1, and (Leyton et al., 2001)].

We additionally described that Thy-1 induces cell migration in astrocytes by interacting not only with integrins but also with Syndecan-4, which plays a known role in cell movement (Avalos et al., 2009). Using mass spectrometry to identify potential Syndecan-4-binding partners, we discovered that PAR-3 was one of the binding proteins, through an interaction dependent on the carboxy-terminal EFYA sequence present on Syndecan-4. When PAR-3 expression was reduced, Thy-1-induced cell migration and FA disassembly were impaired (Valdivia et al., 2020). PAR-3, a protein that plays a central role in establishing and maintaining cell polarity in various cell types (Harris, 2017), was thus defined as a novel adhesome-associated component, with an essential role in FA disassembly during polarized cell migration.

## 3 How mechanobiology has changed our view on cell migration

Cell migration aids in relevant physiological activities, such as embryonic development, morphogenesis, and wound healing. Since living cells are constantly exposed to mechanical stimulation, cell migration is influenced not only by chemical but also by mechanical cues (Mierke, 2020; SenGupta et al., 2021). Thus, forces coming from the extracellular (outside-in) and intracellular (inside-out) environment can control many cellular responses, including migration.

Mechanosensitive receptors expressed at the cell surface interact with the surrounding ECM or receptors on other cells to transduce force toward the inside of the cell. Integrins are considered the classical mechanoreceptors. The engagement of integrins with their ligands induces the formation of FAs and SFs to generate, transmit, and sense mechanical tension (Baker and Zaman, 2010; Schwartz, 2010; Sun et al., 2016). As previously mentioned, 3D super-resolution microscopy has defined FA nanoarchitecture, emphasizing a central force-transduction layer (FTL; see Section 2.1) containing the proteins Talin and Vinculin, which are essential during mechanotransduction because they undergo conformational changes after tension [reviewed in (Kanchanawong et al., 2010; Yan et al., 2015; Haining et al., 2016; Legerstee and Houtsmuller, 2021)]. This intricate FA architecture is tightly and timely controlled. The initial clustering of integrins sequentially recruits proteins such as Talin, Paxillin, and lower levels of Vinculin and FAK, to form NAs that grow into FCs. These FCs are small (< 1 μm^2^; *e.g.*, 0.11 μm^2^ in NIH3T3 cells growing on FN) and unstable since they cannot sustain the tension of the cytoskeleton. Paradoxically, using a tension gauge tether probe that binds αvβ3 integrin, Wang and Ha showed that in epithelial-like cells derived from the ovary (CHO-K1), a tension of 43 pN was required for the initial cell adhesion mediated by FCs (< 15 min after seeding), and that 56 pN was enough to mediate FA and SF formation (∼1 h after seeding) (Wang and Ha, 2013; Austin et al., 2023), supporting the idea that tension is also necessary for FCs to mature into FAs (< 1 μm^2^).

SFs mediate contractility mainly by Myosin II and α-actinin activity. The tension generated by SFs can directly act on FA proteins, regulating their function. Some of these proteins form *catch bonds* (bond lifetime increases with force), others can form *slip bonds*, where the bond lifetime decreases with increasing force, and a third group can form an *ideal bond* that is insensitive to mechanical stress. For instance, selectins and integrins can have a combined catch-slip bond behavior depending on the intensity of the strain that they sense. Experiments using force spectroscopy demonstrated that increasing force first prolonged and then shortened the lifetime of the bond between P-selectin and its ligand, indicating that a catch-to-slip bond switch occurs under force application. The mechanical properties of selectins when binding its ligands are essential for the adhesion of immune cells to the endothelium, and their subsequent detachment from the vessel wall when forces become too high due to blood flow (Sundd et al., 2013; Morikis et al., 2017). Studies using atomic force microscopy and purified α5β1 integrin showed that a tensional force between 10 and 30 pN stabilized the α5β1 integrin-FN interaction lifetime (catch bond); then, when the force was higher than 30 pN, the lifetime of the interaction decreased (slip bond) (Kong et al., 2009). Moreover, Daniel Müller and colleagues also observed a catch-slip transition using live fibroblasts seeded on a FN fragment; however, this catch-slip switch was observed after applying a 40 pN force (Strohmeyer et al., 2017). These observations support the idea that mechanical strain plays a pivotal role in regulating the binding of cell adhesion molecules, such as integrins, with their ligands. Most FA proteins switch their bond behavior when exposed to mechanical forces; therefore, to our knowledge, no examples of ideal bond formation have been described so far for FA proteins.

An asymmetrical strain distribution between the front and rear ends of the cell is crucial for cell migration. Consequently, two tension levels can be identified during migration: those corresponding to non-clustered integrins (FCs, 40 pN), and clustered integrins (FAs, 54 pN). The latter depends on actomyosin and corresponds to high-tension FAs connected to SFs (Wang X. et al., 2015). The disassembly of adhesions occurs at the leading edge on the protrusions during FA turnover and at the rear end after tail retraction. As discussed in the previous Section 2.4, FA disassembly is regulated by various factors, including mechanical tension (Crowley and Horwitz, 1995; Wolfenson et al., 2011). In this context, mechanical tension generated by Myosin II contraction of SFs promotes adhesion maturation, but if this tension is released, FAs disassemble. On the other hand, excessive tension leads to an abrupt loss of adhesion and cell detachment (Sawada et al., 2006; Peacock et al., 2007; Vicente-Manzanares et al., 2007; del Rio et al., 2009; Parsons et al., 2010). Thus, tensional forces are also essential in regulating the dynamic balance between FA formation and disassembly required for cell movement.

Tension can also be sensed and transduced by non-integrin receptors. Some of these receptors can sense shear stress (tangential force of a fluid on a cell layer), as is the case of mechanosensors expressed on the surface of endothelial cells in blood vessels (*e.g.*, Heg-1 and PECAM-1), and other receptors can bind soluble ligands to control integrin activity (*e.g.*, CXCR1 and CXCR2) or bind to the ECM and transduce force. Syndecan-4 has been considered a critical mechanoreceptor for its capacity to interact with the ECM and the actin cytoskeleton. Indeed, the interaction of Syndecan-4 with the HBD of FN induces FA formation (Saoncella et al., 1999; Woods et al., 2000). Consequently, Syndecan-4 deficiency impairs contractility and the formation of mature FAs (Longley et al., 1999; Okina et al., 2012; Cavalheiro et al., 2017). As previously reported, Syndecan-4 clusters in FAs and indirectly binds to FA proteins, such as Paxillin and Hic-5 (Denhez et al., 2002). Moreover, Syndecan-4 has been related to mechanotransduction during shear stress in endothelial cells by mediating diverse physiological effects, such as nitric oxide production, cell adhesion, sensing of the direction of flow, and endothelial cell alignment (Florian et al., 2003; Moon et al., 2005; Baeyens et al., 2014; Nikmanesh et al., 2019). Further studies, such as assays performed with electromagnetic tweezers and elastomeric membranes showed that Syndecan-4 activates mechanotransduction pathways involving ERK phosphorylation and MAPK signaling (Bellin et al., 2009). Similarly, the use of magnetic beads to apply 1 nN of tensional force pulses induced local stiffness, like that produced by integrins (Guilluy et al., 2011; Chronopoulos et al., 2020). Moreover, a constant tension of ∼200 pN applied to Syndecan-4 for 5 min induced the recruitment of Talin-1 and Kindlin-2 in an EGFR-PI3K-dependent manner. RhoA activation timely increased after constant tension, and this effect was blunted after β1 integrin signaling was inhibited by blocking the RGD domains of FN (Chronopoulos et al., 2020). Additional evidence has shown that Syndecan-4 ectodomains could physically interact with integrins (*e.g.*, α3β1, α4β1, α6β4, αvβ3, and αvβ5) and receptor tyrosine kinases (*e.g.*, EGFR, VEGFR2, IGF, and HER2), localize them in FAs and activate signaling pathways related to cell adhesion and migration (Wang H. et al., 2015; Rapraeger, 2021). These results highlight the fact that Syndecan-4 exerts a regulatory and cooperative effect during integrin-based mechanotransduction. Therefore, Syndecan-4 acts as a mechanosensor and regulates cell adhesion and migration. However, even though Syndecan-4 cooperates with integrins to mediate such effects, it is still unknown how the signaling pathways downstream of each receptor converge to potentiate each other.

Although the individual roles of integrins or Syndecan-4 during ECM-driven mechanotransduction have been broadly studied, much less is known about how these receptors sense mechanical cues during cell-cell interactions.

### 3.1 Integrins and Syndecan-4 mechanotransduction during cell-cell interactions

Integrins can mediate mechanotransduction during cell-cell interactions. αLβ2, α4β1, and αvβ3 integrin on the surface of leukocytes can interact with ICAM, VCAM, and PECAM-1 expressed on endothelial cells, respectively (Staunton et al., 1988; Elices et al., 1990; Buckley et al., 1996). For instance, ICAM-1 activation through leukocyte binding promotes cytosolic calcium-mediated myosin activity, leading to endothelial cell contractility. The resulting tension pulls VE-Cadherin in adherens junctions, causing gaps that allow leukocyte transmigration [reviewed in (Schwartz et al., 2021)]. Similarly, αLβ2 (LFA) and αMβ2 integrin in leukocytes can interact with the endothelial junctional adhesion proteins JAM-A and JAM-C, respectively, to allow leukocytes to migrate across the endothelial barrier (Ostermann et al., 2002; Imhof and Aurrand-Lions, 2004; Wojcikiewicz et al., 2009). The interaction force between LFA-1 and Mac-1, and their counterreceptor ligands [ICAMs, JAMs, and receptors for advanced glycation end products (RAGE)] have been extensively studied using single-molecule atomic force microscopy (Zhang et al., 2002; Wojcikiewicz et al., 2006; Yang et al., 2007; Li et al., 2018). In general terms, the strength of the bond depends on the integrin-ligand pair and correlates with the effectiveness of leukocyte adhesion to the endothelium. After attachment, the force is also transduced into the endothelial cells, affecting their contractility and disturbing the stability of tight and adherens junctions to mediate the transmigration of leukocytes through the endothelial layer. Another example is the interaction of leukocyte MAdCAM-1 expressed in high-endothelial venules and endothelial cells in the intestine, with α4β7 (LPAM-1) and α4β1 (VLA-4) integrin in myeloid cells. In this case, the role of MAdCAM-1 is to direct lymphocyte homing by interacting with integrins (Sun et al., 2014; Murakami et al., 2016). Integrins can also bind to receptors on the surface of cancer cells to mediate adhesion to the endothelium. For example, L1CAM on the surface of cancer-stem cell-like cells interacts with αvβ3 integrin in endothelial cells. This interaction promotes the activation of αvβ3 integrin and the phosphorylation of p130Cas and FAK, leading to enhanced migration towards bFGF (Burgett et al., 2016). Overall, these results confirm that integrins need to interact with their counterreceptors in other cells in order to transmit the force that leads to cell migration and invasion.

Less is known about Syndecan-4 mechanotransduction during cell-cell interactions. Although there is evidence that syndecans can interact with receptor tyrosine kinases, the corresponding ligands of these receptors are believed to promote this interaction. For example, VEGFA possesses a highly acidic HBD that allows its binding to the GAG chains of syndecans with different affinities. The strength of the VEGFA-syndecan interaction depends on the nature of the 6-O heparan sulfate chain sulfation, being optimal for Syndecan-2 and very low for Syndecan-1, -3, and -4. Most importantly, VEFGR2 exclusively co-precipitates with syndecans when stimulated with VEGFA. This indirect interaction positively regulates VEGFR2 activity and affects neovascularization and angiogenesis (Echtermeyer et al., 2001; De Rossi et al., 2014; Corti et al., 2019; De Rossi et al., 2021).

Similarly, Syndecan-4 has been involved more recently in regulating VE-cadherin function in the endothelium. In this case, Syndecan-4 interaction with VEGFA triggers the internalization of VE-cadherin, increasing vascular permeability and angiogenesis (De Rossi et al., 2021). Of note, VEGFR2 can also interact with VE-cadherin to regulate mechanotransduction (Coon et al., 2015). Surprisingly, the modulatory effect of Syndecan-4 on this signaling axis has not been studied in the context of mechanobiology, even though VEGFR2 and VE-cadherin both exert an individual and combined role during mechanotransduction in the endothelium (Barry et al., 2015; Coon et al., 2015; de Castro et al., 2015; Dorland and Huveneers, 2017; Mahajan et al., 2017; Miller and Sewell-Loftin, 2021).

**FIGURE 2:**
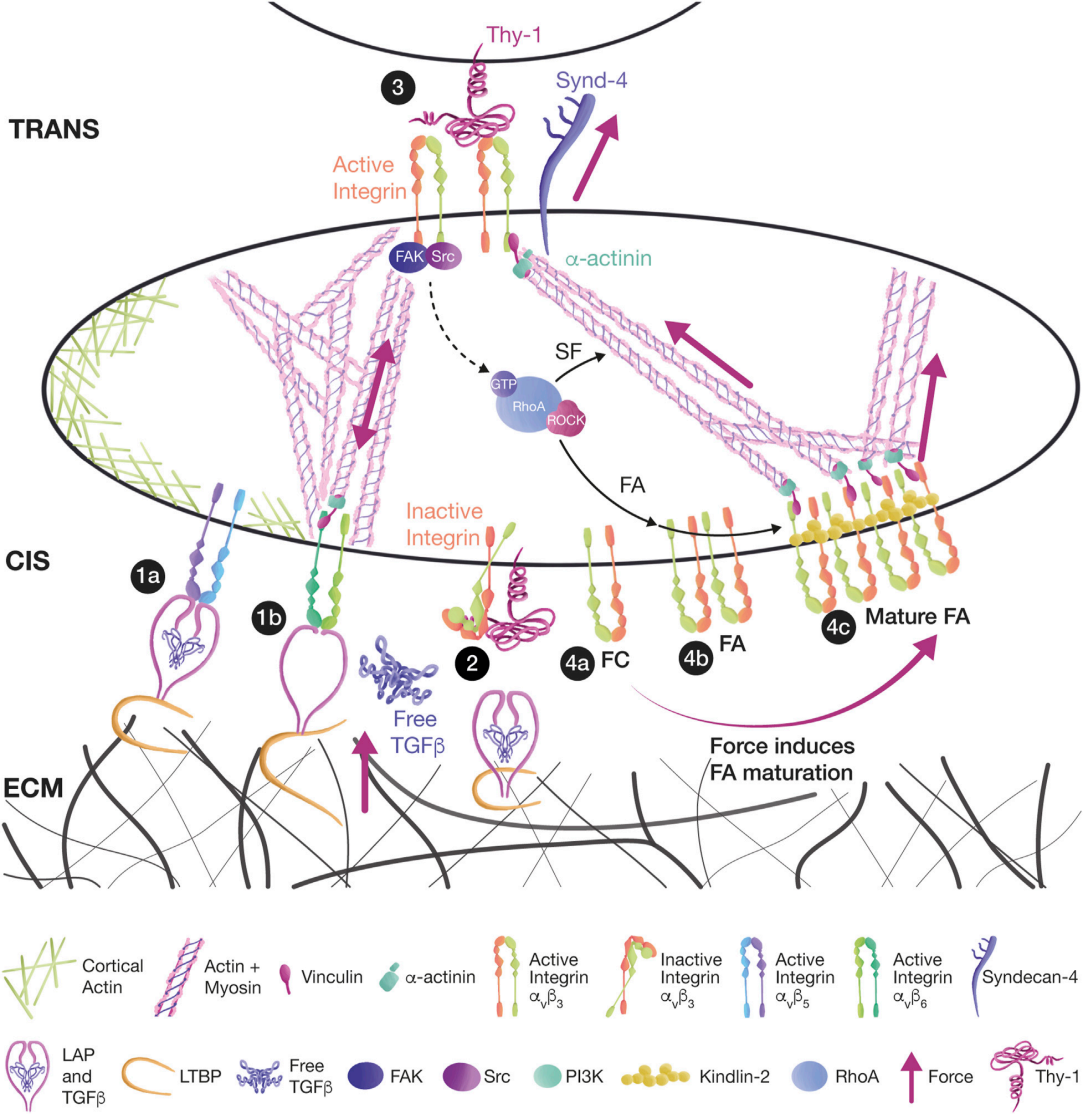
Thy-1 mediates mechanotransduction in *cis and trans,* affecting signaling. **(1a)** Thy-1 binding to αvβ5 and αvβ6 integrins in *cis* has an inhibitory effect during the mechanical activation of TGFβ in myofibroblasts. TGFβ remains dormant by interacting with the latency-associated peptide (LAP) and the latent TGFβ binding proteins (LTBPs). The TGFβ/LAP complex binds to integrins, and LTBPs link TGFβ/LAP to the ECM. **(1b)** Mechanical strain is transduced either from ECM-bound LTBPs or from integrin-bound LAP. Tension releases TGFβ and allows the activation of TGF receptors. **(2)** Thy-1 competes with LAP and ECM for integrin binding, leaving the integrin bent in a low-affinity inactive state in which TGFβ cannot be activated. **(3)** Thy-1 interaction with αvβ3 integrin and Syndecan-4 in *trans* induces the activation of the FAK/Src/RhoA/ROCK signaling pathway, leading to FA and SF formation in astrocytes. **(4a)** FCs are small clusters of integrins. **(4b)** FCs can grow into FAs because of RhoA signaling. **(4c)** Further tension transmitted by SFs leads to FA maturation and Kindlin-2 association.

Another Syndecan-4 receptor is Thy-1. Thy-1 is a unique counterreceptor possessing an RLD peptide that binds to integrin receptors, and an HBD, which interacts with Syndecan-4 (Leyton et al., 2019). Thus, Thy-1 is capable of simultaneously binding and activating integrins and Syndecan-4 to mediate mechanotransduction. This multi-receptor interaction can occur during cell-to-cell interactions (in *trans*) or within the same cells (in *cis*) (Figure 2). We now discuss how mechanobiology has increased the complexity of Thy-1-driven migration.

### 3.2 The Thy-1/integrins/Syndecan-4 interaction in *trans* triggers mechanotransduction

Through its integrin-binding domain (RLD), Thy-1 binds and activates specific integrin heterodimers, including αvβ3, αxβ2, αMβ2, α5β1, and αvβ5 (Saalbach et al., 1999; Leyton et al., 2001; Avalos et al., 2004; Wetzel et al., 2004; Hermosilla et al., 2008; Fiore et al., 2015; Zhu et al., 2018). The engagement of Thy-1 with these integrins has been shown to carry out diverse tension-driven cell functions, such as adhesion, migration, and cell differentiation. For example, Thy-1 expressed on activated microvascular endothelial cells interacts with αvβ3 integrin expressed on melanoma cells to mediate cancer cell invasion. Consequently, Thy-1 knockout mice showed reduced metastasis (Saalbach et al., 2002; Saalbach et al., 2005; Schubert et al., 2013). Moreover, β3 integrin-silenced melanoma cells injected via the tail vein into a syngeneic mouse model exhibited almost undetectable lung tumor mass compared to the wild type cells, in which the tumor mass was 40% of the lung mass (Brenet et al., 2020). Similarly, Thy-1 in endothelial cells also mediated the adhesion and extravasation of leukocytes through αMβ2 (Mac-1) integrin (Wetzel et al., 2004).

Pioneer experiments using a biomembrane force probe to study the mechanical properties of the Thy-1/integrin/Syndecan-4 trimolecular complex revealed that when Thy-1 binds either an integrin (Thy-1/α5β1) or Syndecan-4 (Thy-1/Syndecan-4), the interaction is a classic slip bond. However, the trimolecular bond showed unique mechanical properties: a slip bond with forces < 20 pN, a catch bond from 20 to 35 pN, and then a second slip bond after reaching a tension > 35 pN. This mechanical behavior was described as a “dynamic catch” by the authors, and the proposed mechanism includes an initial α5β1 integrin/Thy-1 interaction that bears most of the force, which after reaching a threshold (∼15 pN), produces bond stiffening, shifting force to the already engaged, but unstretched Syndecan-4. When force induces the extension of the Syndecan-4 GAG chains, both α5β1 integrin and Syndecan-4 can bear a full-force load (Fiore et al., 2014). These results show synergism between integrins and Syndecan-4 during mechanotransduction in a model of melanoma-endothelial cell interaction.

Conversely, in the context of neuron-astrocyte interaction, our group showed a different bond behavior when the trimolecular complex contained αvβ3 integrin. Using molecular tweezers, we found that the Thy-1/αvβ3 integrin and Thy-1/Syndecan-4 interactions showed a slip bond, with forces between 10 and 50 pN. In contrast with Fiore’s findings (Fiore et al., 2014), the Thy-1/αvβ3 integrin/Syndecan-4 complex dissociated faster under tension, suggesting that the slip bond was maintained (Burgos-Bravo et al., 2018). Considering that α5β1 integrin was described to participate in FA maturation and strength, and that αvβ3 integrin can act as a mechanotransductor to regulate migration (Roca-Cusachs et al., 2009; Roca-Cusachs et al., 2012; Fiore et al., 2014; Burgos-Bravo et al., 2018), these reported results allow us to posit a differential role for Syndecan-4 during Thy-1/integrin bond formation. When Syndecan-4 is present, the Thy-1/α5β1 integrin catch bond resists tension to form strong and stable FAs, while the Thy-1/αvβ3 integrin slip bond is less resistant to tension, allowing the dynamic regulation of force sensing and mechanotransduction to facilitate cell migration.

The presence of Syndecan-4 also increased the lifetime of the Thy-1/αvβ3 integrin binary complex both in the absence of force and when force was applied. Remarkably, the lifetime of the Thy-1/αvβ3 integrin interaction was higher in comparison with that of Thy-1/Syndecan-4 after constant force, suggesting that the interaction between Thy-1/Syndecan-4 is weaker than that of the Thy-1/αvβ3 integrin interaction. This can be related to the nature of the interaction, which is protein-protein for Thy-1/αvβ3 integrin and protein-GAG for Thy-1/Syndecan-4 (Burgos-Bravo et al., 2020). However, since in the latter case protein-protein interactions can also occur, this assumption remains to be assessed.

Single-molecule experiments have added substantial evidence of the mechanical properties of the Thy-1/integrin/Syndecan-4 trimolecular complex. However, they may not accurately represent the full view since receptor clustering is important for FA and SF formation in astrocytes. For instance, although Thy-1 immobilized on a plate acts as a cell substrate or soluble recombinant Thy-1-Fc protein can induce weak cell adhesion and signaling, stimulation with Thy-1-Fc conjugated to Protein A-sepharose beads successfully stimulates strong cell adhesion, RhoA activation, SF formation, and migration in astrocytes (Avalos et al., 2004; Avalos et al., 2009; Kong et al., 2013). Similarly, co-culture experiments using neuronal cells expressing Thy-1 on their surface can also stimulate cytoskeletal changes that lead to astrocyte migration (Kong et al., 2013), which were also observed after stimulating astrocytes with EL4 cells, a lymphoma-derived cell line that expresses high levels of Thy-1 (Leyton et al., 2001). These observations sustain the idea that multivalent Thy-1 conjugated to beads or Thy-1 present on the surface of other cells can successfully stimulate strong adhesion, SF formation, and migration of cells, suggesting that clustering of Thy-1 with its co-receptors is necessary for an efficient cellular response.

Certainly, as with all GPI-anchored proteins, Thy-1 localizes in lipid rafts, and its interactions in c*is* could relocate integrins in lipid rafts (Barker et al., 2004; Hudon-David et al., 2007; Wang et al., 2010). Similarly, the aggregation of integrins in nanodomains has been linked to their activity (Lietha and Izard, 2020). However, it is still not entirely understood whether integrin clustering is triggered after ligand binding, leading to outside-in integrin activation, or whether clustering occurs due to integrin activation. Nevertheless, experiments performed with RGD peptides suggest that both possibilities are feasible (Ginsberg et al., 2005; Yu et al., 2011) and propose that integrin clustering occurs in phases. First, the initial binding of RGD to integrins leads to the recruitment of more integrins, Talin, Paxillin, and FAK, by lateral diffusion. Subsequently, actin polymerization and contractility mediated by Myosin generate an inward force associated with distant integrin clusters, resulting in larger FAs and the stimulation of lamellipodium extension (Yu et al., 2011). Accordingly, our group showed in primary astrocytes that αvβ3 Integrin overexpression by transfection or TNFα treatment induces integrin microclusters without triggering integrin activation, but priming the cells to respond to Thy-1 stimulation (Lagos-Cabre et al., 2018).

**FIGURE 3:**
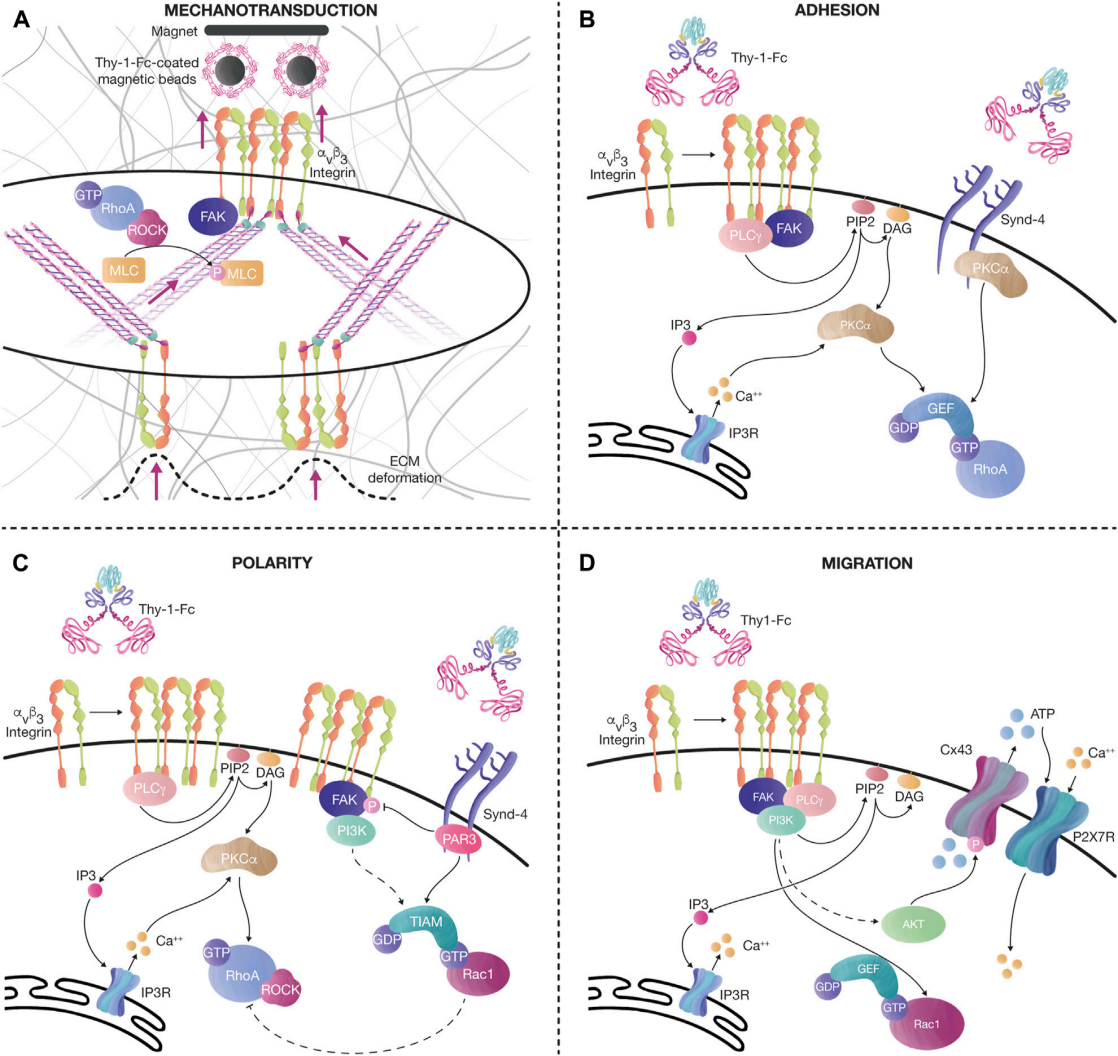
Thy-1 effects on mechanotransduction, cell adhesion, polarity, and migration. **(A)** Tension increases the levels of αvβ3 integrin at the cell surface after pulling magnetic beads coated with recombinant Thy-1-Fc. This effect is followed by an increase in cell contractility, evidenced by the augmented number and thickness of SFs, and elevated phosphorylation of MLC, probably due to the activation of the FAK/RhoA/ROCK signaling axis, as previously described. The tension transduced through SFs also induces ECM deformation. **(B)** Thy-1 interacts with αvβ3 integrin and Syndecan-4 to stimulate FA formation (< 15 min). Integrin clustering recruits signaling molecules, such as FAK and PLCγ. PLCγ activity on PIP2 produces DAG and IP3. IP3 activates Ca^++^ release from the IP3R in the endoplasmic reticulum. Ca^++^, together with DAG, activates PKCα, leading to RhoA activation. Alternatively, PKCα can also be activated by interacting with the variable region of the Syndecan-4 cytoplasmic tail, synergizing RhoA activity. **(C)** Longer stimulation of αvβ3 integrin and Syndecan-4 with Thy-1-Fc (< 60 min), induces changes in cell polarity, FA disassembly, and migration. The polarity complex protein Partitioning defective 3 (PAR-3) binds to the Syndecan-4 cytoplasmic tail to inhibit FAK phosphorylation and activate the RacGEF, Tiam, which further mediates Rac1 activation. PI3K is also activated after sustained stimulation with Thy-1-Fc in astrocytes, stimulating the activation of Rac1. Moreover, once Rac1 is active, an inhibitory axis can decrease RhoA activity through p190RhoAGAP. Altogether, this signaling pathway helps the cell disassemble FAs and migrate in a directional manner. **(D)** Prolonged Thy-1-Fc stimulation (∼16 h) allows the cell to migrate. Ca^++^ is released from the IP3R at the endoplasmic reticulum by IP3 produced by PLCγ. FAK/PI3K/Akt activation downstream of integrins induces Rac1 activation and phosphorylation of Connexin43 (Cx43) channels, which release ATP to the extracellular compartment. ATP activates the P2X7 receptor to mediate Ca^++^ influx. Bursts of cytoplasmic Ca^++^ transients, together with active Rac1, stimulate cell migration.

Moreover, *in vitro* assays using magnets to pull magnetic beads coated with recombinant Thy-1-Fc have shown that mechanical strain increases integrin levels at the cell surface, an effect that is followed by an increase in contractility reflected by elevated phosphorylation of MLC and an increase in the number and thickness of SFs (Perez et al., 2021) (Figure 3A).

Our previous work using Thy-1-Fc beads was based on stimulating cells on the dorsal surface to effectively form FAs and SFs on the ventral part of the cells (Perez et al., 2021). However, how exactly does dorsal stimulation transduce the signals to the interior of the cell to generate an effect on the ventral surface? We speculate that dorsal engagement of integrins triggers signaling pathways that involve the activation of small Rho GTPases. These pathways would then crosstalk with those engaged by the ventral integrins stimulated by the ECM-cell contacts, thus enhancing/synergizing the effect, and producing a more robust cellular response (Figure 3A).

### 3.3 The Thy-1/integrin/Syndecan-4 interaction in *cis* regulates mechanotransduction

Primary fibroblasts expressing Thy-1 (Thy-1^+/+^) form strong FAs and SFs and, consequently, are less migratory than fibroblasts not expressing this protein (Thy-1^−/−^). Thy-1 expressed on the surface of lung fibroblasts interacts in *cis* with αvβ5 integrin, keeps it in a low-affinity bent conformation, and decreases its binding to ECM proteins (Zhou et al., 2010). Moreover, the Thy-1/αvβ3 integrin interaction occurs in lipids rafts, where Fyn and Src are recruited to FAs. Fyn itself has been described as critical during mechanosignaling since it activates the RhoA pathway by reducing Src-induced phosphorylation of p190RhoGAP through the recruitment of CBP and by directly phosphorylating RhoGEFs (Guilluy et al., 2011; Fiore et al., 2015).

Additionally, Thy-1 can also regulate mechanotransduction by modulating the avidity of the mechanoreceptors and modifying the composition of the ECM. The mechanics and composition of the ECM are particularly important during idiopathic pulmonary fibrosis, where a stiffer ECM is associated with mature fibrosis. Here, fibroblasts sense mechanical cues and undergo aberrant activation (Guo et al., 2022). In this context, a stiff FN-rich provisional ECM promotes FA formation and actomyosin contractility, leading to fibroblast-induced remodeling of the ECM, thereby amplifying the profibrotic phenotype. Consequently, pulmonary fibroblasts secrete FN independently of Thy-1 expression; however, Thy-1^+/+^ fibroblasts produce two-to three-fold more collagen than Thy-1^−/−^ cells, contributing to the deposition of a softer ECM (Derdak et al., 1992; Abeysinghe et al., 2005). Moreover, in a model of fibrosis, Fiore et al. showed that fibrosis-associated fibroblasts lacking Thy-1 expression displayed elevated activation of αvβ3 integrin, thus leading to enhanced fibroblast activation, stiffer provisional ECM, and progression of fibrosis (Fiore et al., 2018).

The inhibitory effect of Thy-1 binding to integrins in *cis* has also been associated with the mechanical activation of TGFβ. The latency-associated peptide (LAP) binds TGFβ, and latent TGFβ binding proteins (LTBPs) link the TGFβ/LAP complex to the ECM, keeping a dormant TGFβ pool that can be activated by mechanical force transduced by αvβ5 and αvβ6 integrins expressed in myofibroblasts. Tension transmitted either from ECM-bound LTBPs or from myofibroblast contractility can pull LAP, release TGFβ, and allow the activation of TGF receptors (Munger et al., 1998; Annes et al., 2002; Annes et al., 2004; Wipff et al., 2007). Thy-1 competes with LAP and the ECM for integrin binding; thus, Thy-1 interaction with αvβ5/αvβ6 integrins in *cis* maintains these integrins inactive, and TGFβ cannot be activated, thus affecting myofibroblast differentiation and fibrosis progression (Figure 2) (Zhou et al., 2004; Zhou et al., 2010).

### 3.4 What we still do not know about Thy-1/integrin/Syndecan-4 mechanotransduction

Syndecan-4 mechanical properties have been broadly studied in the context of shear stress and ECM-driven mechanotransduction. To date, only a few studies have shown the mechanical properties of the Thy-1/Syndecan-4 and Thy-1/integrins/Syndecan-4 interactions (Fiore et al., 2015; Burgos-Bravo et al., 2018; Burgos-Bravo et al., 2020; Perez et al., 2021). Studying the effects of the Thy-1/integrin/Syndecan-4 trimolecular complex in the context of mechanotransduction is important since mechanical strain controls many physiological and pathological functions, such as embryonic development, morphogenesis, wound healing, cancer, and atherosclerosis.

Even though Syndecan-4 cooperates with integrins during mechanotransduction, it is still not clear how the signaling between both receptors synergizes. We posit the possibility of downstream signaling crosstalk between integrins and Syndecan-4, in which integrin activation would induce the activation of RhoA and several kinases, including FAK and PI3K. Similarly, the activation of Syndecan-4 would also recruit FAK and PKC and further activate RhoA, increasing the overall response (Figure 3B). Optimal signaling would occur when both receptors are active since the loss of either receptor has shown defects in contractility and FA formation (Saoncella et al., 1999; Bass et al., 2007). Another possibility is the direct *cis* interaction between integrins and Syndecan-4. It is plausible that they influence each other’s membrane location; therefore, external engagement of either integrins or Syndecan-4 could favor receptor clustering and potentiate the initial signaling.

Syndecan-4 could also antagonize integrin signaling. After sustained Thy-1 stimulation (∼30 min), PAR-3 is recruited to the Syndecan-4 cytoplasmic tail, leading to Rac1 activation through its GEF, Tiam (Valdivia et al., 2020). Rac1 signaling antagonizes RhoA activity, prompting the cell to polarize, disassemble FAs, decrease contractility, and migrate (Figure 3C). In this case, it is still unknown how Syndecan-4 signaling turns off integrin signaling. However, it is likely that Rac1 activates p190RhoGAP and induces RhoA inactivation, as previously described [reviewed in (Lawson and Burridge, 2014)] (Figure 3C). Thus, the crosstalk between integrin and syndecan receptors can tightly regulate FA dynamics and the cytoskeleton changes occurring during cell adhesion and migration.

Alternatively, Syndecan-4 probably acts as a regulator of the inside-out activation of integrins. In this scenario, we can hypothesize that Syndecan-4 engagement could activate certain signaling pathways, such as PI3K or PKCα, which would then transduce signals to activate integrins and enhance cellular contractility. A similar mechanism has already been proposed for the mechanoreceptor PECAM-1. In this case, shear stress activates PECAM-1, leading to the PI3K signaling necessary for β1 integrin activation (Collins et al., 2012). A different scenario has been described in fibroblasts, where Src-mediated Syndecan-4 phosphorylation on Y^180^ inactivates Arf6, which regulates integrin recycling (Morgan et al., 2013). In this case, Syndecan-4 phosphorylation lowers α5β1 integrin recycling to the membrane, while favoring the passage of αvβ3 integrin from endosomes to the plasma membrane. This is another type of integrin switch but mediated by Syndecan-4 signaling, which is crucial for controlling integrin trafficking and FA dynamics and inducing FA stabilization and more directional cell migration by inactivating Arf6 (Morgan et al., 2013).

## 4 What we have learned from our studies on cell adhesion and migration

### 4.1 The discovery of the integrin/Thy-1 interaction in cell adhesion and migration

Our interest in this topic started a little over 20 years ago while searching for the ligand of the cell adhesion molecule Thy-1. Research on Thy-1 led to the important discovery that GPI-anchored glycoproteins could signal intracellularly despite only crossing the outer leaflet of the plasma membrane and lacking a transmembrane-spanning domain (Morris, 1992). In addition, Thy-1 was known to modulate neurite outgrowth (Tiveron et al., 1992); however, this and other potential functions of Thy-1 were uncertain due to its unidentified ligand.

We were the first group to describe that the RLD tripeptide found in the Thy-1 sequence recognized the αvβ3 integrin present in astrocytes (Leyton et al., 2001; Hermosilla et al., 2008). Integrin ligation by Thy-1 was functional and induced the formation of FAs in astrocytes, an effect that was precluded when the cells were stimulated with a Thy-1 fusion protein in which the RLD tripeptide was mutated to RLE (D^18^E). Other features of this interaction that supported a role for the αvβ3 integrin were that the adhesion of astrocytes to Thy-1 required Mn^2+^ or Mg^2+^ but neither Ca^2+^ nor a combination of both Ca^2+^/Mg^2+^, and that the interaction was inhibited by anti-β3 but not by anti-β1 integrin antibodies (Hermosilla et al., 2008). The FAs formed because the Thy-1-engaged integrin interaction possessed many proteins described in IACs (see Section 2.1), including Vinculin, Paxillin, p130Cas, and FAK (Leyton et al., 2001). Our initial findings reporting αvβ3 integrin as a Thy-1 receptor were later confirmed in other cell types, including melanomas and breast cancer cells (Brenet et al., 2020), fibroblasts (Valdivia et al., 2020), and leukocytes (Wetzel et al., 2004). The αvβ3 integrin/Thy-1 interaction was additionally extended to other integrins, such as αxβ2 and αMβ2 in leukocytes (Choi et al., 2005), and α5β1 in melanoma cells (Fiore et al., 2015)[reviewed in (Leyton et al., 2019)].

### 4.2 Focal adhesions induced by the direct interaction of Thy-1 with integrins/Syndecan-4

As previously mentioned, FAs are formed and maintained by the activation of RhoA, whose activity is also required to form SFs (Ridley and Hall, 1992). In astrocytes stimulated with Thy-1, we have shown that integrin clustering, RhoA activation, and enhanced activity of the RhoA effector, ROCK, are required to promote FA and SF formation in cells bound to their own secreted matrix (Avalos et al., 2002; Avalos et al., 2004) (Figure 3B). Thus, this mechanism for cell contraction seems to be conserved whether generated by cell-cell [Thy-1/integrin (Avalos et al., 2004; Hermosilla et al., 2008)] or cell-matrix [integrin/ECM (Berrier and Yamada, 2007)] interactions.

In our original study, results obtained by competing the Thy-1/integrin interaction with antibodies, recombinant proteins, or peptides suggested that a third molecule was involved, since complete inhibition was never attained (Leyton et al., 2001). Considering the available information, we hypothesized that Syndecan-4 could act as a co-receptor for αvβ3 integrin to respond to Thy-1. This hypothesis was additionally supported by studies indicating the existence of a direct interaction between Thy-1 and sulfated glycans (Hueber et al., 1992). By using different heparin dilutions, we demonstrated that Thy-1-stimulated adhesion of astrocytes involved a Thy-1-HBD interaction, which was required for Syndecan-4 binding, as demonstrated by silencing Syndecan-4 or expressing a mutant that lacked its cytoplasmic domain (Avalos et al., 2009). In addition, treatment of astrocytes with heparitinase decreased the number of FAs formed, and the observed contacts were of smaller size (Avalos et al., 2009). Moreover, mutation of the basic amino acids composing the HBD (REKRK→AEAAA) led to the production of a recombinant protein that could no longer stimulate FA formation. Thus, both the integrin-binding domain (RLD) and the HBD (REKRK) of Thy-1 were necessary to trigger astrocyte adhesion (Avalos et al., 2009). We additionally demonstrated that Thy-1-mediated integrin/Syndecan-4 engagement involved the activation of PKCα upstream of the loading of RhoA with GTP (active state) (Avalos et al., 2009) (Figure 3B). Because RhoA is associated with cell contraction and strong cell adhesion, we proposed that Thy-1/integrin interaction would prevent cell migration. However, we then discovered that upon sustained stimulation with Thy-1 (24 h), RhoA activity decreased, whereas that of Rac1 increased, thus leading to astrocyte migration (Kong et al., 2013).

### 4.3 Signaling mechanisms involved in Thy-1-induced astrocyte migration

PKCα is a calcium- and DAG-dependent enzyme; therefore, we next studied signaling mechanisms that could account for changes in these second messengers. Over the years, we have been able to decipher, in part, the puzzle of the signal transduction pathways involved in Thy-1/integrin-induced astrocyte adhesion and migration. The elucidated sequence of events includes the activation of FAK/Src/PI3K/PLCγ and hydrolysis of PIP2 to generate DAG and IP3, which activates the IP3R and induces intracellular calcium increase (Kong et al., 2013). Most recently, we have shown that AKT, activated downstream of PI3K, phosphorylates and opens the hemichannel Connexin43, releasing ATP (Perez-Nunez et al., 2023) (Figure 3D). The opening of Connexin43 is accompanied by Pannexin1 hemichannel activation, and both hemichannels release ATP to the extracellular compartment, thereby leading to the activation of the purinergic receptor P2X7R and allowing calcium entry (Henriquez et al., 2011; Alvarez et al., 2016) (Figure 3D). This functional link between integrins and P2X7R in the context of cell migration was a novel finding exhibited by the DITNC1 astrocyte cell line but later, was also found in breast cancer and melanoma cells (Brenet et al., 2020).

Remarkably, the signaling events described in the DITNC1 astrocyte cell line in response to Thy-1 were not observed in primary astrocytes unless these cells were treated with inflammatory cytokines (Lagos-Cabre et al., 2017). TNF-treated primary astrocytes showed increased levels of reactivity markers, such as GFAP, iNOS, and Connexin43. To our surprise, astrocytes responded to Thy-1 stimulation only when they were reactive (TNF-treated). The response comprised morphological features associated with *in vivo* reactivity, such as hypertrophy, a fibroblast-like phenotype, and increased cell migration (Lagos-Cabre et al., 2017). The molecular mechanisms triggered by Thy-1 in these reactive astrocytes were similar to those reported for the DITNC1 cell line, and the inflammatory signals were necessary to increase the levels of both αvβ3 integrin and Syndecan-4 (Lagos-Cabre et al., 2017; Palacios et al., 2023). Importantly, the increase in αvβ3 integrin levels occurred in a NFkB-dependent manner (Lagos-Cabre et al., 2017; Palacios et al., 2023).

Most importantly, αvβ3 integrin appeared in large membrane patches in immunofluorescence microscopy assays, suggesting that TNF prompted the cells to respond more effectively to Thy-1 by increasing the surface levels of this integrin and inducing the formation of αvβ3 integrin microclusters (Lagos-Cabre et al., 2018). Astrocytes overexpressing β3 integrin exhibited spontaneous microclusters in the absence of TNF treatment and responded to Thy-1 without any prior pro-inflammatory exposure (Lagos-Cabre et al., 2017). These results were intriguing, and further analysis indicated that the sole overexpression of this integrin was sufficient to turn the astrocytes into reactive ones and prompt their migration in response to Thy-1 stimulation (Lagos-Cabre et al., 2017; Lagos-Cabre et al., 2018).

Another central question that interested us was: How does the ATP that travels through open hemichannels reach the juxtamembrane localization to be released by the cells? Based on the reported evidence and our results indicating that exocytosis was not involved in ATP release [Brefeldin A did not affect Thy-1-induced ATP release (Alvarez et al., 2016)], we hypothesized that the release of ATP required calcium uptake by mitochondria and re-localization of these organelles to the cell cortex via microtubule-dependent transport. Before we were able to test this assumption with our astrocyte cell line, different researchers showed that migrating cells transfer the mitochondria to the leading edge over the microtubules in order to drive ATP production and provide energy demands where needed (Cunniff et al., 2016). These results were confirmed in cancer and mesothelial cells, as well as fibroblasts, and therefore, we anticipated that astrocytes probably use a similar mechanism to provide the local ATP production required for its release, although this has not been demonstrated yet using astrocytes. ATP could then be released through hemichannels, for example, via the opening of Connexin43 phosphorylated by active AKT (Perez-Nunez et al., 2023).

The role of Syndecan-4 on astrocyte migration was also intriguing, and therefore, we studied its role by generating a Thy-1 fusion protein mutated in the HBD (Thy-1(AEAAA)-Fc). We learned that this mutated protein does not promote astrocyte migration (24 h), despite inducing ATP release, although in lower amounts (40%), and in a delayed manner (15 min instead of 10 min), compared to ATP release induced by the wild type protein Thy-1(RLD)-Fc (Alvarez et al., 2016). On the contrary, the Thy-1 fusion protein mutated in its integrin-binding domain (Thy-1(RLE)-Fc) did not induce cell migration, nor elicited any ATP release. However, Thy-1(RLE)-Fc induced astrocyte migration when added with a threshold amount of BzATP (a non-hydrolyzable ATP analogue added at a concentration three orders of magnitude lower than that required to induce migration). Our results suggested that Thy-1-engaged αvβ3 integrin led to ATP release, whereas Thy-1-ligated Syndecan-4 was likely involved in the regulation of the kinetics, as well as the threshold amount of ATP released by astrocytes required to induce cell migration (Alvarez et al., 2016). These results led us to propose a sequential activation: integrins first, followed by Syndecan-4, which would then be in charge of inducing a feed-forward loop to maintain the cycle of protein activation required for cells to move.

In agreement with the idea of Syndecan-4 being a regulator of the αvβ3 integrin/Thy-1 interaction, we then reported that Syndecan-4 forms a ternary complex with Thy-1 and αvβ3 integrin, increasing the lifetime of the Thy-1/αvβ3 integrin bond even when exposed to force, as would occur with mechanical signals coming from the ECM or cell-to-cell contacts (Burgos-Bravo et al., 2018). Importantly, the use of optical tweezers allowed us to demonstrate that at a single molecule level, the Thy-1/αvβ3 integrin bond dissociated faster by force application (Burgos-Bravo et al., 2018). However, when Syndecan-4 was present, bond stabilization of the bimolecular Thy-1/integrin interaction accelerated the biological functions exerted by αvβ3 integrin (Burgos-Bravo et al., 2018). We provided additional validation of these findings by simultaneously applying chemical and mechanical stimulation to astrocytes and found that the kinetics of astrocyte adhesion and contraction were accelerated by adding a mechanical force to Thy-1 stimulation (Perez et al., 2021). Considering these results, we proposed that Syndecan-4 leads to structural changes in αvβ3 integrin, allowing it to interact more efficiently with Thy-1, thus inducing integrin clustering and activation, and a faster cellular response.

An additional piece of the signaling puzzle was added after we discovered that upon Thy-1 binding, Syndecan-4 interacted with PAR-3, a protein that had not been associated with the pathways that determine whether a cell migrates or not. In one of our recent reports (Valdivia et al., 2020), we described that after Thy-1 stimulation, PAR-3 interaction with the Syndecan-4 intracellular tail is required for the disassembly of FAs through FAK dephosphorylation and the activation of Tiam1 and Rac1 (Figure 3C). This report is relevant because it is the first one to describe PAR-3 as an adhesome-associated component involved in the polarized migration of astrocytes and fibroblasts through the regulation of FA dynamics (Valdivia et al., 2020).

Therefore, Thy-1 signaling triggered in astrocytes by binding to its partners, αvβ3 integrin and Syndecan-4, is an intricate network regulated by chemical (Thy-1) and mechanical (force) cues (Figure 3A) that requires a threshold level of receptors, which are controlled in part, by inflammatory signals.

## 5 Concluding remarks and clinical relevance

In this review, we summarized studies related to adhesion receptors, their signaling mechanisms and how the complex networks they form are regulated by the ECM; we also described the mechanical forces generated by the ECM, and the interactions with other cells. The main adhesion receptors are integrins and Syndecan-4, which are also mechanosensors and thus, translate the mechanical cues to the cytoskeleton and change the cellular responses. Integrins and the hundreds of proteins that are recruited to cell adhesion points, which change from NAs to FCs, and then to FAs and mature FAs, are the main components of the adhesome, a structure that has become relevant due to the control it exerts on cell adhesion and migration.

Cell adhesion and migration are cyclic and dynamic processes highly regulated by the intricate interconnections that occur within the adhesome. Many proteins that form part of the adhesome are activated/inactivated, switched on/off, or phosphorylated/dephosphorylated, and these events are constantly occurring within the complex to maintain the dynamics and cyclicity of these processes. During FA dynamics, the cytoskeleton also plays a key role; both actin filaments and microtubules contribute to regulate FA maturation and disassembly to maintain the cyclic machinery that controls cell migration. In the past years, much has been learned about the composition of the adhesome and the turnover of FAs; however, how much of this knowledge could be applied to living organisms is still a matter of debate. The current availability of 3D culturing techniques, organoids, organs on a chip, etc., will be key to solving this discussion. Similarly, the comparative analysis of adhesomes present in normal *versus* tumoral and metastatic cells can help identify the components of the adhesome that are differentially expressed in diverse stages of cancer, which could lead to the development of targeted therapies (Sauzay et al., 2019; Brenet et al., 2020; Keller-Pinter et al., 2021; Bergonzini et al., 2022).

Mechanobiology has certainly added complexity to how we initially pictured cell migration. Mechanical strain is tightly regulated at the level of substrate stiffness, mechanoreceptor abundance, the readiness of mechanotransducers to signal, and ECM remodeling. Our results have shown that the interaction between Thy-1 and integrins/Syndecan-4 in *trans* can regulate FA and SF formation, enhancing the complexity of integrin-mediated mechanotransduction.

Acute or chronic inflammation is a hallmark of many pathological conditions, such as cancer, wound healing, autoimmune diseases, and arteriosclerosis. Different pathophysiological processes are positively or negatively affected by mechanical forces. Of relevance, Thy-1, integrins, and Syndecan-4 expression can be stimulated by inflammatory molecules, which may play an important role in regulating conditions of clinical relevance, such as wound healing [reviewed in (Perez et al., 2022b)]. Accordingly, our group and others have shown that Thy-1 and Syndecan-4 promote skin wound healing (Echtermeyer et al., 2001; Araki et al., 2009; Bass et al., 2011; Brooks et al., 2012; Vuong et al., 2015; Das et al., 2016; Perez et al., 2022a). In particular, we demonstrated that Thy-1 increases the healing rate by increasing skin perfusion. However, further experiments are necessary to determine if the interaction between Thy-1, Syndecan-4, and integrins is relevant during wound remodeling and scarring, where mechanical forces play a pivotal role.

Cell migration is necessary but requires to be tightly regulated. If occurring in an uncontrolled manner, cell migration is detrimental, as occurs during cancer metastasis, autoimmune diseases, or fibrosis. The interaction of Thy-1 with integrins in *cis* plays an essential role in inhibiting TGFβ signaling and fibrosis. Indeed, in an *in vivo* model of lung fibrosis, the administration of soluble Thy-1 therapeutically inhibits integrin-mediated fibrosis (Phuan et al., 2019). Our discoveries have contributed to developing transcendental pre-clinical research, and we expect that these studies can soon translate into therapeutics for curing or improving the outcome of many clinical conditions related to cell adhesion and migration.

## Abbreviations

bFGF, Basic fibroblast growth factor; CD90, Cluster of differentiation 90; ECM, Extracellular matrix; EGF, Epidermal growth factor; FA, Focal adhesion; FAK, Focal adhesion kinase; FC, Focal contact; FGFR, Fibroblast growth factor receptor; FN, Fibronectin; GAGs, Glycosaminoglycans; GAP, GTPase-activating protein; GEF, Guanine nucleotide exchange factor; GDI, Guanine nucleotide dissociation inhibitor; HBD, Heparin-binding domain; IAC, Integrin-associated complex; IGF, Insulin growth factor; LAP, Latency-associated peptide; LTBP, Latent TGFβ binding protein; MAPK, Mitogen-activated protein (MAP) kinase; NA, Nascent adhesion; PAR-3, Partition-defective 3; PDGF, Platelet-derived growth factor; PLC, Phospholipase C; SF, Stress fiber; TGFβ, Transforming growth factor β; Thy-1, Thymus cell antigen 1; VEGF, Vascular endothelial growth factor.
